# Direct association between diet and the stability of human atherosclerotic plaque

**DOI:** 10.1038/srep15524

**Published:** 2015-10-22

**Authors:** Isabel Gonçalves, Elisavet Andersson Georgiadou, Sören Mattsson, Göran Skog, Luís Pedro, José Fernandes e Fernandes, Nuno Dias, Gunnar Engström, Jan Nilsson, Kristina Stenström

**Affiliations:** 1 Cardiovascular Research Group, Department of Clinical Sciences, Malmö, Lund University, Sweden; 2 Department of Cardiology, Skåne University Hospital, Malmö, Sweden; 3 Lund University, Department of Physics, Division of Nuclear Physics, Lund, Sweden; 4 Lund University, Department of Clinical Sciences Malmö, Medical Radiation Physics, Skåne University Hospital, Malmö, Sweden; 5 Lund University, Department of Geology, Radiocarbon Dating Laboratory, Sweden; 6 Department of Vascular Surgery, Santa Maria Hospital, Faculty of Medicine, University of Lisbon, Portugal; 7 Departments of Haematology and Vascular Diseases, Skåne University Hospital, Malmö, Sweden; 8 Department of Clinical Sciences, Malmö, Lund University, Sweden

## Abstract

Mediterranean diet has been suggested to explain why coronary heart disease mortality is lower in southern than northern Europe. Dietary habits can be revealed by isotope ratio mass spectrometry (IRMS) measurement of carbon (δ^13^C) and nitrogen (δ^15^N) in biological tissues. To study if diet is associated with human plaque stability, atherosclerotic plaques from carotid endarterectomy on 56 patients (21 Portuguese and 35 Swedish) were analysed by IRMS and histology. Plaque components affecting rupture risk were measured. Swedish plaques had more apoptosis, lipids and larger cores, as well as fewer proliferating cells and SMC than the Portuguese, conferring the Swedish a more rupture-prone phenotype. Portuguese plaques contained higher δ^13^C and δ^15^N than the Swedish, indicating that Portuguese plaques were more often derived from marine food. Plaque δ^13^C correlated with SMC and proliferating cells, and inversely with lipids, core size, apoptosis. Plaque δ^15^N correlated with SMC and inversely with lipids, core size and apoptosis. This is the first observational study showing that diet is reflected in plaque components associated with its vulnerability. The Portuguese plaques composition is consistent with an increased marine food intake and those plaques are more stable than those from Swedish patients. Marine-derived food is associated with plaque stability.

Coronary heart disease (CHD) mortality differs markedly across Europe and is generally lower in the southern than in the northern and eastern parts of the continent^1^. Although the underlying causes for this difference remain to be clarified, there is emerging evidence that the Mediterranean diet contributes to the lower CHD mortality in southern Europe^2^,^3^,^4^,^5^. Most acute coronary events are caused by thrombotic occlusion on top of a ruptured atherosclerotic plaque. The risk for plaque rupture is dependent on the structure of the plaque and rupture-prone or vulnerable plaques are characterized by enhanced inflammation, extensive lipid accumulation, large necrotic core, as well as loss of fibrous tissue and of the connective tissue-producing smooth muscle cells^6^,^7^,^8^,^9^,^10^,^11^,^12^.

To what extent dietary habits can influence CHD risk by direct effects on atherosclerotic plaque structure is not known. Using isotope ratio mass spectrometry (IRMS) it is possible to estimate the dietary origin of molecular components incorporated into biological tissues by analysing the composition of certain isotopes^13^,^14^. The relative abundance of stable isotopes of carbon (expressed as δ^13^C) and nitrogen (δ^15^N) can be used to differentiate between different types of terrestrial and marine-derived food (Fig. 1a).

To understand the role of diet in CHD mortality, we investigated the dietary origin of the nitrogen and carbon molecules present in atherosclerotic plaques obtained from Portuguese and Swedish carotid surgery patients. We also analysed how the dietary origin of the plaque nitrogen and carbon molecules related to structural components associated with plaque stability.

## Results

There were no significant differences in clinical characteristics between Portuguese and Swedish patients, except that the use of statins was more common in Swedish patients and the time between the clinical event and operation that was shorter in the Portuguese patients (Table 1).

We first analysed the structural components important for plaque vulnerability in both Portuguese and Swedish plaques. Swedish plaques were found to have more apoptotic cells, increased levels of lipids and larger cores than Portuguese plaques (Fig. 2a). The Swedish plaques were also characterized by fewer proliferating cells and less smooth muscle cells (Fig. 2b). Figure 2a depicts destabilizing components analysed, namely apoptosis, lipids and core size. The higher levels present in plaques from Swedish patients are shown in blue boxes as compared to the Portuguese in red. Accordingly, using the same colour scheme, in Fig. 2b, the stabilizing plaque components measured are shown, e.g. less smooth muscle cells and proliferating cells in the Swedish plaques.

In Fig. 3 are shown two representative images of carotid plaques from the two countries where larger amounts of lipids making a large lipid core (Fig. 3a, in red) is seen in the Swedish plaques. Moreover, reduced number of smooth muscle cells is observed in the consecutive section (Fig. 3b, in brown). In contrast, a lipid-poor and smooth muscle cell-rich plaque with a thick cap from a Portuguese subject is shown in Fig. 3c,d. All of these features are compatible with a more vulnerable phenotype of the Swedish plaques.

**Figure 1:**
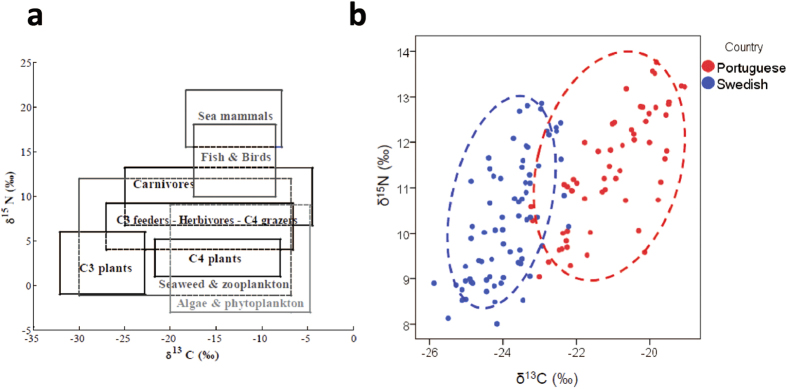
(**a**) Generalized isotopic trophic diagram for terrestrial and marine food webs^34^. (**b**) Stable isotope (δ^13^C and δ^15^Ν) diagram for Portuguese and Swedish plaques.

**Figure 2:**
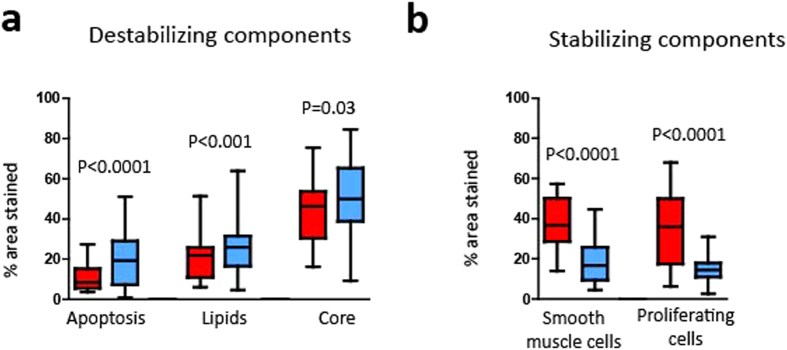
Boxplots showing the histological analysis of carotid plaque components (% area) from Portuguese (in red) and Swedish (in blue) patients: (**a**) apoptosis (TUNEL, P < 0.0001), lipids (Oil Red O, P < 0.001) and core (P = 0.03), (**b**) smooth muscle cells (alpha-actin, P < 0.0001) and proliferative cells (PCNA, P < 0.0001).

**Figure 3:**
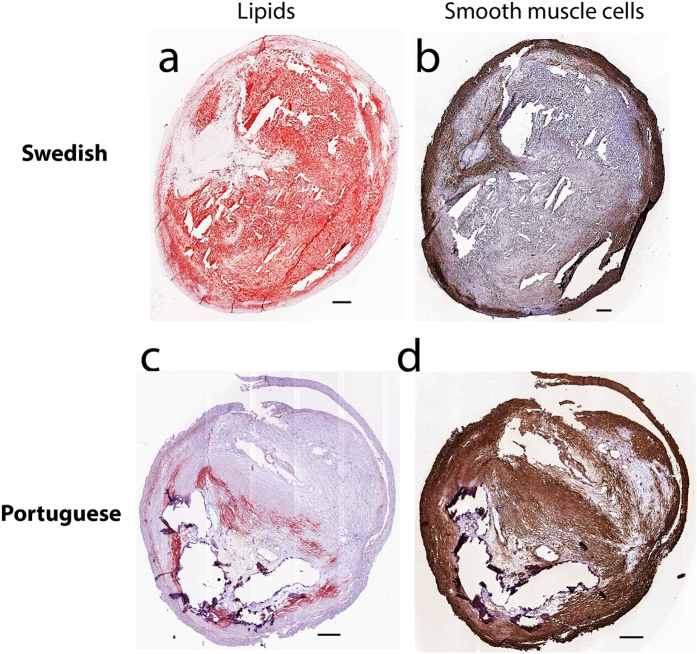
Representative images of a Swedish (**a,b**) and a Portuguese (**c,d**) carotid plaque stained for lipids (in red, Oil Red O; left panel) and for smooth muscle cells (SMCs, in brown, alpha-actin; right panel). Scale bar 500 μm.

**Figure 4:**
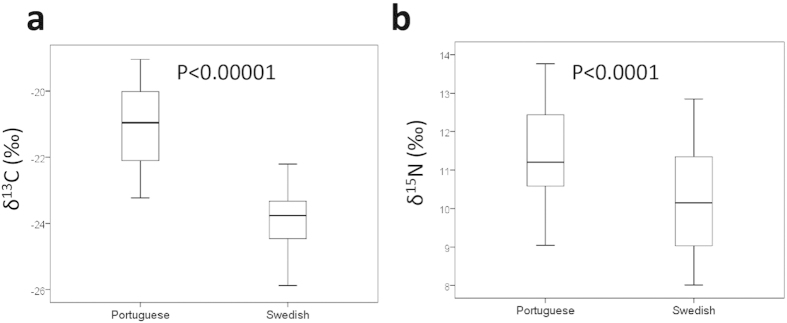
Boxplots showing (**a**) δ^13^C levels (P < 0.00001) and (**b**) δ^15^Ν levels in carotid plaques from Portuguese and Swedish patients (P < 0.0001).

The distribution of δ^13^C and δ^15^N isotopes in Swedish (in blue) and Portuguese (in red) plaques demonstrated markedly different patterns (Fig. 1b). Comparison of δ^13^C and δ^15^N levels showed that plaques from Portuguese patients contained significantly higher values of both the δ^13^C and δ^15^N than plaques from Swedish patients (Fig. 1b and 4). Both δ^13^C and δ^15^N levels correlated positively with staining for smooth muscle cells while inverse associations were noted for lipids, core size, apoptosis (Table 2). δ^13^C was also positively correlated with PCNA staining (Table 2). All associations remained significant after adjustments for age, gender, country of origin, current smoker and diabetes (Table 2).

## Discussion

We show for the first time that diet is reflected in plaque components related with its stability. We used mass spectrometry to analyse carbon and nitrogen isotope ratios in human atherosclerotic plaques and expressed the isotopic fractionation in terms of δ^13^C and δ^15^N. The distribution of these isotopes in living tissues reflects their dietary origin with high levels of both reflecting marine dietary sources, particularly fish (Fig. 1a)^15^,^16^,^17^,^18^,^19^,^20^,^21^. We detected increased levels of both δ^13^C and δ^15^N in human carotid plaques from Portuguese patients compared to Swedish. Portuguese plaques had a more stable phenotype and δ^13^C and δ^15^N levels correlated significantly with several factors associated with atherosclerotic plaque stability including the lipid content, core size, smooth muscle cells, as well as rates of cell proliferation and apoptosis. These associations remained significant when adjusting for country of origin demonstrating that a high intake of seafood was associated with a more stable plaque phenotype in both countries.

There is a wide range of possible speculations for the differences in mortality between southern and northern Europe^22^, including differences in diet and in plaque stability. The detailed dietary patterns of the two countries studied are well characterized in the reports of Food and Agriculture Organization of the United Nations. The Portuguese, on average, consume more fish products (13.4%) than Swedes (7.8%)^23^. As shown in Fig. 1a, the Portuguese diet includes more fish (leading to higher δ^13^C and δ^15^N values) and maize (C4 group, meaning higher δ^13^C values), than the Swedish diet, which in general contains a significant amount of potatoes (C3 group, leading to lower values of both δ^13^C and δ^15^N), as well as other C3 foodstuffs (such as wheat, oats, rice, etc.). The δ^13^C and δ^15^N values now measured in the plaque samples further support this epidemiologic data.

It is well documented that the majority of acute cardiovascular events are caused by rupture of atherosclerotic plaques with a thin fibrous cap. Morphological examinations have demonstrated these plaques are characterized by inflammatory infiltrates (primarily macrophages), large necrotic cores, extensive intra- and extra-cellular lipid accumulation, cell death and reduced smooth muscle repair capacity^7^,^8^,^9^,^10^,^12^,^24^. In our sample collection, carotid plaques from Portuguese patients have a more stable histological phenotype than the Swedish, showing less apoptosis, lower lipid content, smaller cores, as well as more proliferating cells and smooth muscle cells, that stabilize the fibrous cap, avoiding rupture and the development of symptoms. Nation-related differences in plaque structure have previously been reported in autopsy studies^25^,^26^. It could be argued that the concept of the vulnerable plaque as the major cause of acute cardiovascular events is based mainly on circumstantial retrospective observations. However, data from the PROSPECT study^27^, as well as combined data from the Oxford Plaque Study and AtheroExpress^6^ have provided prospective data confirming that presence of vulnerable plaques are associated with an increased risk of future cardiovascular events.

There are some differences between the patient cohorts that could affect our findings. (1) The time of collection (Portuguese plaques 2000–2001 vs. Swedish plaques 2005–2011), (2) the time between the clinical event and surgery (shorter in Portugal than in Sweden) and (3) the use of statins (more common in Swedish patients). Data from the AtheroExpress biobank have shown that atherosclerotic plaques obtained in 2010–2011 had a more stable phenotype than plaques obtained in 2001–2002^28^. Accordingly, it is unlikely that the more stable phenotype of Portuguese plaques in the present study could be explained by the year of surgery. Also the shorter time between the clinical event and surgery for the Portuguese plaques is unlikely to explain their more stable phenotype because there is less time for healing responses after plaque rupture to occur. Finally, statins are known to have stabilizing effects on plaque structure^29^, thereby one would expect the Swedish plaques to be more stable than the Portuguese.

Our results using samples from Portugal and Sweden should be interpreted with caution, as they cannot be directly extrapolated to other regions of Europe. Large multinational studies ideally evaluating the characteristics of the plaques in asymptomatic patients through Europe are now required. Our study cannot provide exact amounts of dietary elements or even exact types of maritime/seafood dietary sources. The IRMS results also have to be interpreted with some caution, as the δ^13^C and δ^15^N values do not only reflect the diet, but also depend on the type of tissue^17^,^30^. Finally, the histological analysis was only performed in the most stenotic region of the plaques. Plaques are heterogeneous and plaque structure can vary along the vessel wall. The most stenotic region is considered to have most of the representative components^31^, but variations in other parts of the plaque are possible. Finally the plaques studied corresponded to advanced atherosclerotic disease. No conclusions can be extrapolated to subjects with normal arterial walls or less advanced stages of the disease.

Taken together, these data indicate that the atherosclerotic plaque composition is consistent with the intake of certain dietary atoms. The composition of the Portuguese atherosclerotic plaques is associated with the increased intake of seafood and those plaques are more stable than those from Swedish patients.

This study is pioneer in showing that marine-derived food is associated with plaque stability. Despite the need for further studies, the ultimate clinical implication of this knowledge is to encourage a simple and cheap strategy, as marine dietary intake in prevention of atherosclerosis, to stabilize rupture-prone plaques that ultimately lead to myocardial infarction and stroke.

## Methods

### Clinical samples

We studied human atherosclerotic plaques obtained by carotid endarterectomy from 56 patients: 21 Portuguese and 35 Swedish. The Swedish patients underwent carotid surgery at the Skåne University Hospital, Malmö, Sweden, during 2005 to 2011 and the Portuguese were operated in 2000–2001 at the Cardiovascular Institute of Lisbon, Lisbon, Portugal. The patients’ characteristics are described in Table 1. All patients were preoperatively assessed by an independent neurologist as having significant stenosis (stenosis >70% for the plaques associated with symptoms (transient ischemic attacks (TIA), strokes or amaurosis fugax) or >80% for the asymptomatic). Stenosis grade was measured according to the velocity criteria assessed by ultrasound^32^.

All methods were carried out in accordance with the approved guidelines. The study was approved by the local ethical committee (Regional Ethical Review Board in Lund). All patients gave informed consent.

### Sample processing

All plaques were snap-frozen in liquid nitrogen at endarterectomy. Two consecutive 1-mm-thick transverse sections of the most stenotic region were sliced from each sample, one for δ^13^C/δ^15^N analysis and one for histology. The plaque samples for the isotope fractionation analysis were dissected into different regions (fibrous cap, core and interface between the core and the outer cleavage plan of the plaque towards the media).

### δ^13^C and δ^15^N measurements

The samples were dried and prepared as previously described^33^. The stable isotope (δ^13^C, δ^15^N) analysis was performed at IRMS facility at the Environmental Isotope Laboratory (EIL) at University of Waterloo, Ontario, Canada. The required weight of each sample was 0.25-0.30 mg. The ratios of the samples were calibrated against several different standards of known isotopic composition. The analytical precision obtained for the standards was <0.3‰ for N and <0.2‰ for C (1σ)^34^.

### Histological and immunohistochemical analysis

The fragments were cryosectioned in transversal 8 μm sections, fixed with Histochoice (Amresco, Ohio, USA), dipped in 60% isopropanol and in 0.4% Oil Red O in (60%) isopropanol (for 20 min) to stain lipids. For macrophage assessment, primary monoclonal antibody mouse anti-human CD68, clone KP1 (DakoCytomation, Glostrup, Denmark), diluted in 10% rabbit serum 1:100, and secondary antibody biotinylated polyclonal rabbit anti-mouse, rabbit F(ab´)2 (DakoCytomation, Glostrup, Denmark), dilution 1:200 in 10% of rabbit serum, were used. For smooth muscle cells (alpha-actin), primary antibody monoclonal mouse anti-human smooth muscle actin clone 1A4 (DakoCytomation, Glostrup, Denmark), diluted in 10% rabbit serum 1:50, and secondary antibody biotin rabbit anti-mouse Ig (DakoCytomation, Glostrup, Denmark), dilution 1:200 in 10% of rabbit serum, were used. To visualize apoptotic cells in plaques, TUNEL (terminal deoxynucleotidyl transferase dUTP nick-end labelling) *In Situ* Cell Death detection kit POD (Roche Applied Science, Indianapolis, Ind, USA) was used, according to manufacturer’s instructions. Proliferation was assessed by staining with mouse monoclonal [PC10] anti-human proliferating-cell nuclear antigen (PCNA) proliferation marker (ab29) (1:100, Abcam, Cambridge, UK; overnight incubation at 4 °C). Sections were subsequently incubated with biotinylated polyclonal rabbit anti-mouse F(ab)2 (E0413,1:200, DakoCytomation, Glostrup, Denmark) for 30 minutes and then with peroxidase-labelled streptavidin (Vectastain ABC-AP kit, Vector Laboratories, Peterborough, UK). Measurements of the area of plaque (% area) for the lipids, macrophages, smooth muscle cells, apoptosis and proliferation, as well as the core region were quantified blindly using Biopix Q 2.1.8 (Gothenburg, Sweden) after scanning with ScanScope Console Version 8.2 (LRI imaging AB, Vista CA, USA).

### Statistics

The distribution of δ^13^C and δ^15^N was approximately normal (skewness <0.33) and no log transformation was applied. Values are presented as mean (standard deviation, SD). Two-group comparisons were performed with Chi-square or Mann-Whitney test. Spearman’s rho test was used for correlation analysis. Multiple linear regressions, with δ^13^C or δ^15^N as dependent variables, were used to adjust the relationship between histologic plaque components and δ^13^C and δ^15^N, for potential confounding factors. Age, gender, current smoker, diabetes, country of origin and histological plaque component were entered into the regression model. We also investigated whether presence of symptoms, time between symptom and operation, statin use, degree of stenosis and date of the operation, could act as confounding factors for the relationship between isotope pattern and histological plaque components. However, these factors showed no substantial effect on this relationship and were therefore not included in the final regression model. Differences were considered statistically significant at P < 0.05. SPSS 21 (SPSS Inc., Chicago, Ill, USA) has been used for statistical analysis.

## Additional Information

**How to cite this article**: Gonçalves, I. *et al.* Direct association between diet and the stability of human atherosclerotic plaque. *Sci. Rep.* **5**, 15524; doi: 10.1038/srep15524 (2015).

## Figures and Tables

**Table 1:** Clinical characteristics of the patients that underwent carotid endarterectomy. SD, standard deviation; NS, non-significant.

	Swedish patients operated 2005-2011 (n = 35)	Portuguese patients operated 2000-2001 (n = 21)	P value
Age (years)	73.7 (SD 9.7)	69.0 (SD 10.6)	NS
Gender (males)	23 (66%)	16 (76%)	NS
Symptoms	27 (77%)	13 (62%)	NS
Time between symptoms and operation (days)	29.2 (SD 29.4)	10.0 (SD 10.8)	0.005
Degree of stenosis (%)	87 (SD 9.8)	83 (SD 7.3)	NS
Type 2 diabetes	16 (46%)	6 (29%)	NS
Hypertension	26 (74%)	20 (95%)	NS
Smoking (currently)	12 (34%)	4 (19%)	NS
Statin use	22 (63%)	5 (24%)	0.006
Fasting lipoproteins (mmol/L):			
Cholesterol	4.4 (SD 1.2)	5.3 (SD 1.4)	NS
Low-density lipoprotein (LDL)	2.7 (SD 1.0)	2.9 (SD 1.4)	NS
High-density lipoprotein (HDL)	1.1 (SD 0.4)	0.9 (SD 0.4)	NS
Triglycerides	1.5 (SD 0.8)	1.5 (SD 0.2)	NS

**Table 2:** Correlations between the stable isotope ratios δ^13^C and δ^15^N and the different human atherosclerotic plaque components assessed histologically and immunohistochemically. NS, non-significant.

Area of component	Non-adjusted		Adjusted*	
	δ^13^C	δ^15^N	δ^13^C	δ^15^N
Core	−0.323^§^	−0.311^§^	−0.186^†^	−0.272^‡^
Lipids (Oil Red O)	−0.41^§^	−0.256^‡^	−0.328^§^	−0.186^†^
Apoptosis (TUNEL)	−0.516^§^	−0.374^§^	−0.457§	−0.328^§^
Macrophages (CD68)	NS	0.201^†^	NS	NS
Smooth muscle cells (alpha-actin)	0.769^§^	0.437^§^	0.361^§^	0.227^‡^
Proliferating cells (PCNA)	0.423^§^	NS	−0.188^†^	NS

^*^Adjusted for age, gender, country, current smoker and diabetes. ^†^P < 0.05; ^‡^P < 0.01; ^§^P < 0.0001.
